# Shift work and occupational stress among nurses: Its impact on physical and mental health

**DOI:** 10.1192/j.eurpsy.2026.12037

**Published:** 2026-07-31

**Authors:** K. Rachubińska, O. Charkiewicz, D. Schneider-Matyka, E. Grochans, A. Derezińska, A. Cybulska

**Affiliations:** Department of Nursing, Pomeranian Medical University in Szczecin, Szczecin, Poland

## Abstract

**Introduction:**

Shift work disrupts circadian rhythms, leads to sleep disorders, and complicates the balance between professional, family, and social life. For nurses, this challenge is intensified by the constant need for vigilance, rapid decision-making in stressful situations, and responsibility for patients’ lives, all of which contribute to high occupational stress and adverse health outcomes.

**Objectives:**

The study aimed to assess the relationship between shift work and stress levels among nurses and to evaluate the impact of occupational stress on their health.

**Methods:**

The study included 224 nurses and male nurses employed at three hospitals in Szczecin, Poland. Data were collected using an original questionnaire and standardized instruments: the Expanded Nursing Stress Scale (ENSS), the Perceived Stress Scale (PSS-10), and the Depression, Anxiety, and Stress Scale (DASS-21).

**Results:**

According to the PSS-10, most respondents reported moderate (36.28%) or high (42.92%) stress levels, while the DASS-21 indicated normal severity of depressive, anxiety, and stress symptoms. The ENSS identified the most stressful factors as workload, discrimination, and conflicts with physicians and supervisors. Stress was most strongly associated with responsibility for patient health (63.72%), aggressive behavior of patients and families (62.83%), and fear of committing irreversible errors (60.18%). A majority (91.15%) confirmed that workplace stress affects their health, with 66.81% reporting negative consequences such as back pain, insomnia, fatigue, and low mood. No statistically significant associations were found between stress severity and sociodemographic variables. However, work experience, education, place of residence, and workplace were significant predictors of occupational stress. Generational differences were also observed, with Generation Z reporting higher stress levels than Millennials, particularly in relation to interactions with patients.

**Conclusions:**

Nurses working shifts are highly exposed to occupational stressors, which adversely affect both physical and mental health. Sociodemographic and occupational factors, including work experience, education, and generation, significantly influenced perceived stress. The findings emphasize the need for effective organizational strategies and time management interventions to mitigate stress and protect nurses’ wellbeing.

**Disclosure of Interest:**

None Declared

